# Supplementary material for: Detection of correlated hidden factors from single cell transcriptomes using Iteratively Adjusted-SVA (IA-SVA)
Source: Sci Rep. 2018 Nov 19;8:17040. doi: 10.1038/s41598-018-35365-9 (PMC6242813; doi:10.1038/s41598-018-35365-9)
Supplement: Supplementary file 1 — Supplementary Information [file 41598_2018_35365_MOESM1_ESM.pdf]

Detection of correlated hidden factors from single cell transcriptomes using Iteratively Adjusted-SVA (IA-SVA)

Supplementary Information

Donghyung Lee[1,*], Anthony Cheng[1,2], Nathan Lawlor[1], Mohan Bolisetty[3], and Duygu Ucar[1,2,4,*]

[1] The Jackson Laboratory for Genomic Medicine, Farmington, 06032, CT, USA, [2] Department of Genetics and Genome Sciences, University of Connecticut Health Center, Farmington, 06030, CT, USA, [3] Bristol-Myers Squibb, Pennington, NJ, 08534, USA, [4] Institute of Systems Genomics, University of Connecticut Health Center, Farmington, 06030, CT, USA.

* Correspondence: *donghyung.lee@jax.org* and *duygu.ucar@jax.org*

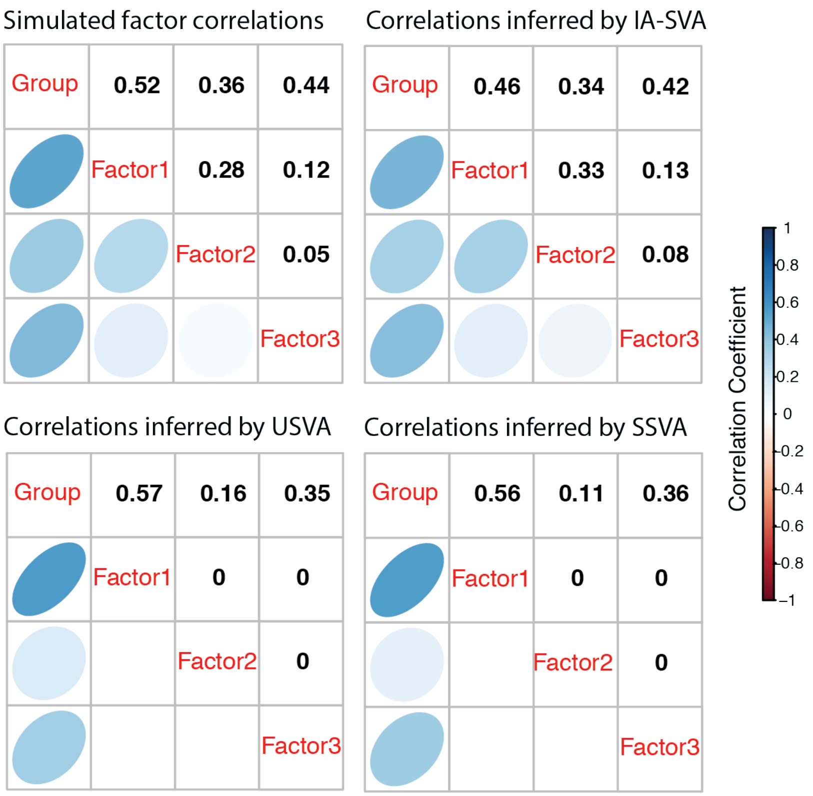

Figure S1. **Correlation structure among true and estimated factors (Group, Factor1, Factor2 and Factor3) and the group variable based on simulated scRNA-seq data.** We studied the true correlation structure (Pearson correlation coefficient) among all simulated factors (Group, Factor1, Factor2 and Factor3) and compared this against the correlation structure based on detected factors. IA-SVA accurately estimated correlations between the group variable and hidden factors, whereas SVA methods failed to do so particularly for the correlations between three hidden factors due to their orthogonality assumption.

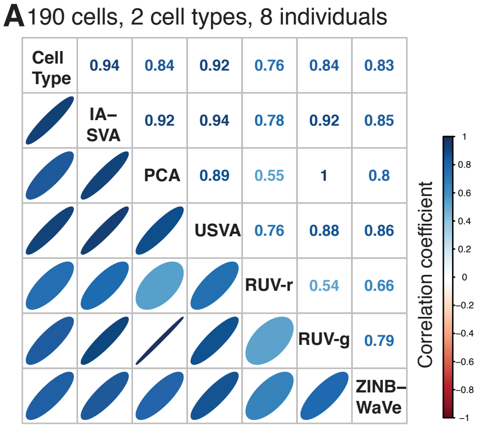

B Summary of 100 runs

		IA-SVA	PCA	USVA	RUV-r	RUV-g	ZINB-WaVe
Correlation	m	0.926	0.853	0.855	0.726	0.839	0.813
	sd	0.013	0.021	0.035	0.049	0.050	0.017
Time (s)	m	12.61	0.141	1.216	76.078	75.748	162.611
	sd	1.607	0.026	0.263	7.778	7.710	30.084

* Except PCA, tissue + library-size + sample-ids are used as covariates
* m = mean, sd = standard deviation, s = seconds
* For each method, the highest correlated SV is used among the top 3.
* For IA-SVA, permutation test is used with 20 permutations

Figure S2. Comparison of alternative methods for discovering the SV associated with the (known) cell type assignment. (**A**) We compared IA-SVA's performance to PCA, unsupervised SVA, RUV-r, RUV-g, and ZINB-WaVe using scRNA-seq data from human cortex. IA-SVA outperformed other algorithms at uncovering the SV associated with cell types. (**B**) The comparison is repeated 100 times by selecting 1000 genes for each run. Average correlation scores and run times are denoted for each algorithm along with the standard deviations. ZINB-WaVe was the slowest among of all tested methods.

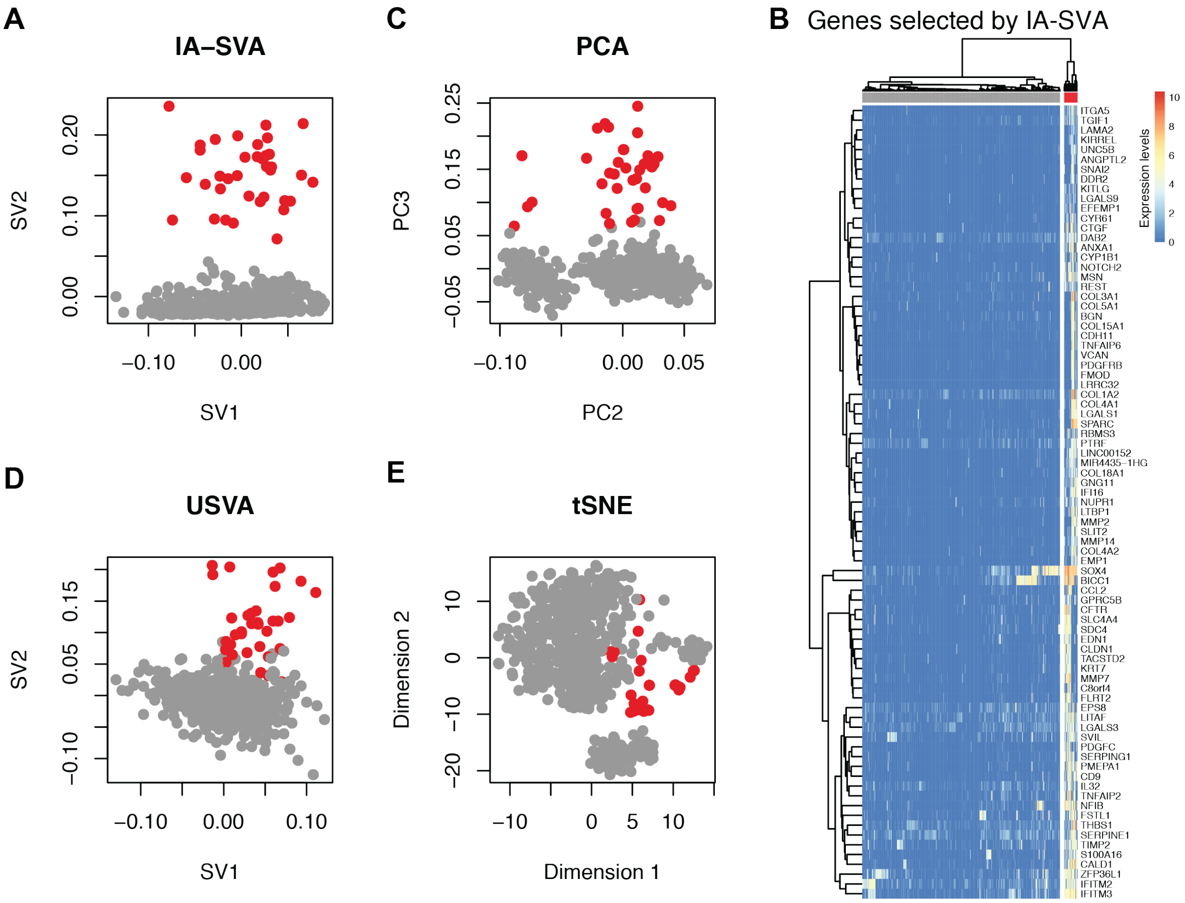

Figure S3 IA-SVA recapitulates detected heterogeneity in alpha cells in a second pancreatic islet scRNA-seq data. (**A**) Outlier alpha cells captured using IA-SVA and same cells marked in respective (**C**) PCA, (**D**) USVA, and (**E**) tSNE analyses. Cells are clustered into two groups (red vs. gray dots) based on IA-SVA's surrogate variable 2 (SV2 > 0.05). (**B**) Hierarchical clustering (ward.D2 and cutree_cols =2) of alpha cells using 81 genes significantly associated (FDR < 0.05 and R^2 > 0.3) with SV2. 36 cells clearly separate from the rest of the cells based on their high expression of these genes. The values in the heatmap and the color bar are log-transformed (base e) normalized counts, i.e., $\log_e$ (1 + read counts normalized using SCnorm). In PCA, PC1 was disregarded since it maps to the geometric library size. While PCA, USVA and tSNE detected some heterogeneity among alpha cells, they failed to clearly separate these 36 cells. PCA and tSNE captured clusters originated from known factors (e.g., 'Patient ID'), which are adjusted for in IA-SVA and USVA.

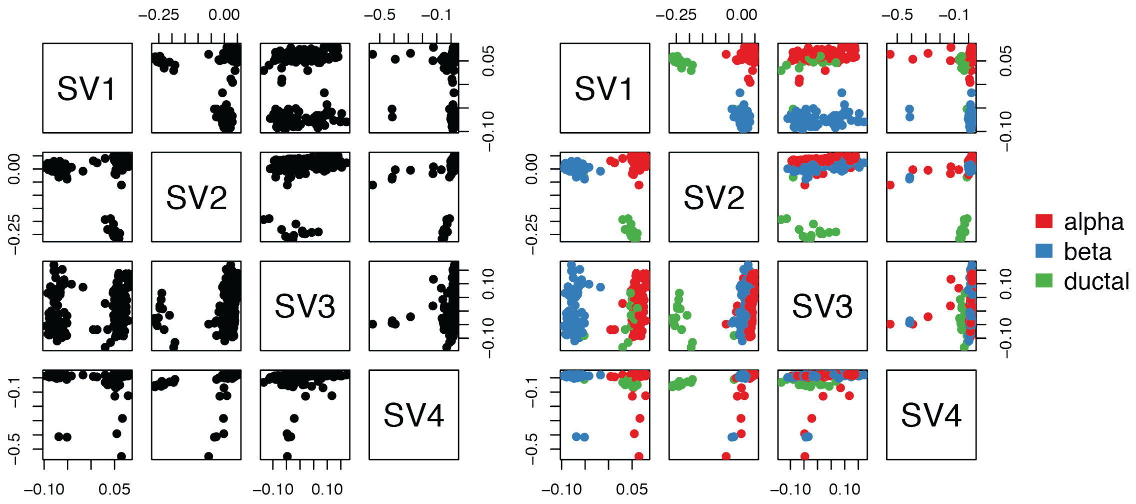

Figure S4. **Pairwise scatter plot of top four significant IA-SVA surrogate variables (SV) detected from human islet scRNA-Seq data including three cell types: alpha (GCG), beta (INS) and ductal (KRT19) cells.** Cells on the right subfigure are color-coded based on the original assignment. SV1 and SV2 clearly separate cells into distinct clusters, therefore are good candidates for further analyses. GO enrichment and pathway analyses results of 92 genes associated (FDR < 0.05, $R^2 > 0.5$) with SV1 and SV2 are illustrated in Supplementary Table S4. SV4 captures technical heterogeneity stemming from cell contamination (e.g., stacked doublets), which was observed in **Figure 2** and **Figure S3**. Go enrichment and pathway analyses results of 94 genes associated (FDR < 0.05, $R^2 > 0.3$) with SV4 are illustrated in Supplementary Table S6.

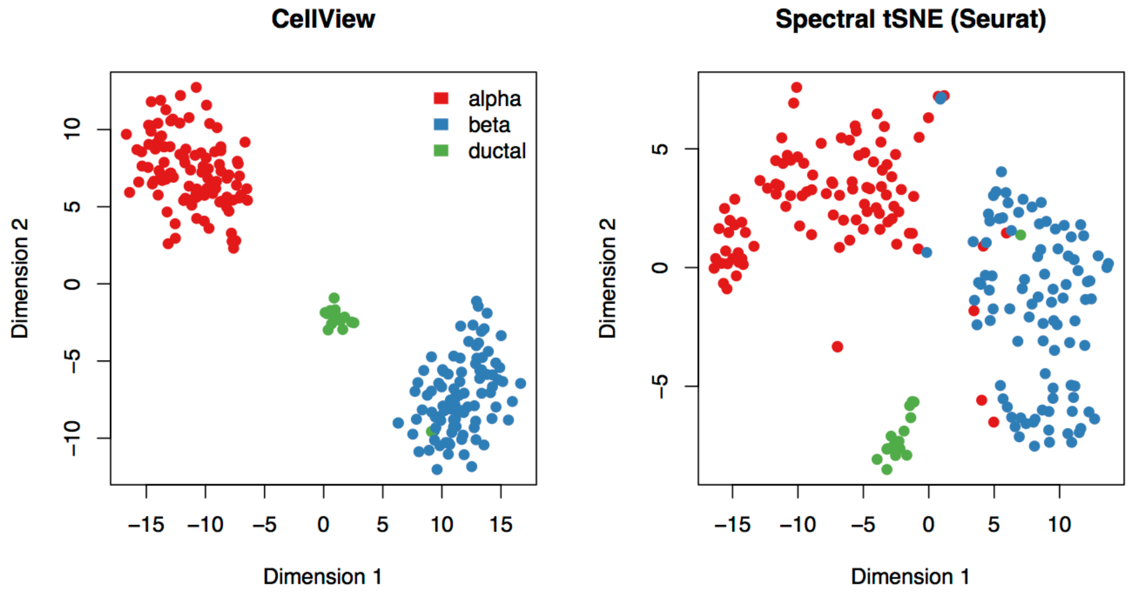

Figure S5. **tSNE analyses using 1000 most over-dispersed genes (CellView, left) and using significant PCs obtained from highly over-dispersed genes detected Seurat (Spectral tSNE, right)**. Cells are color-coded based on the original cell-type assignment.

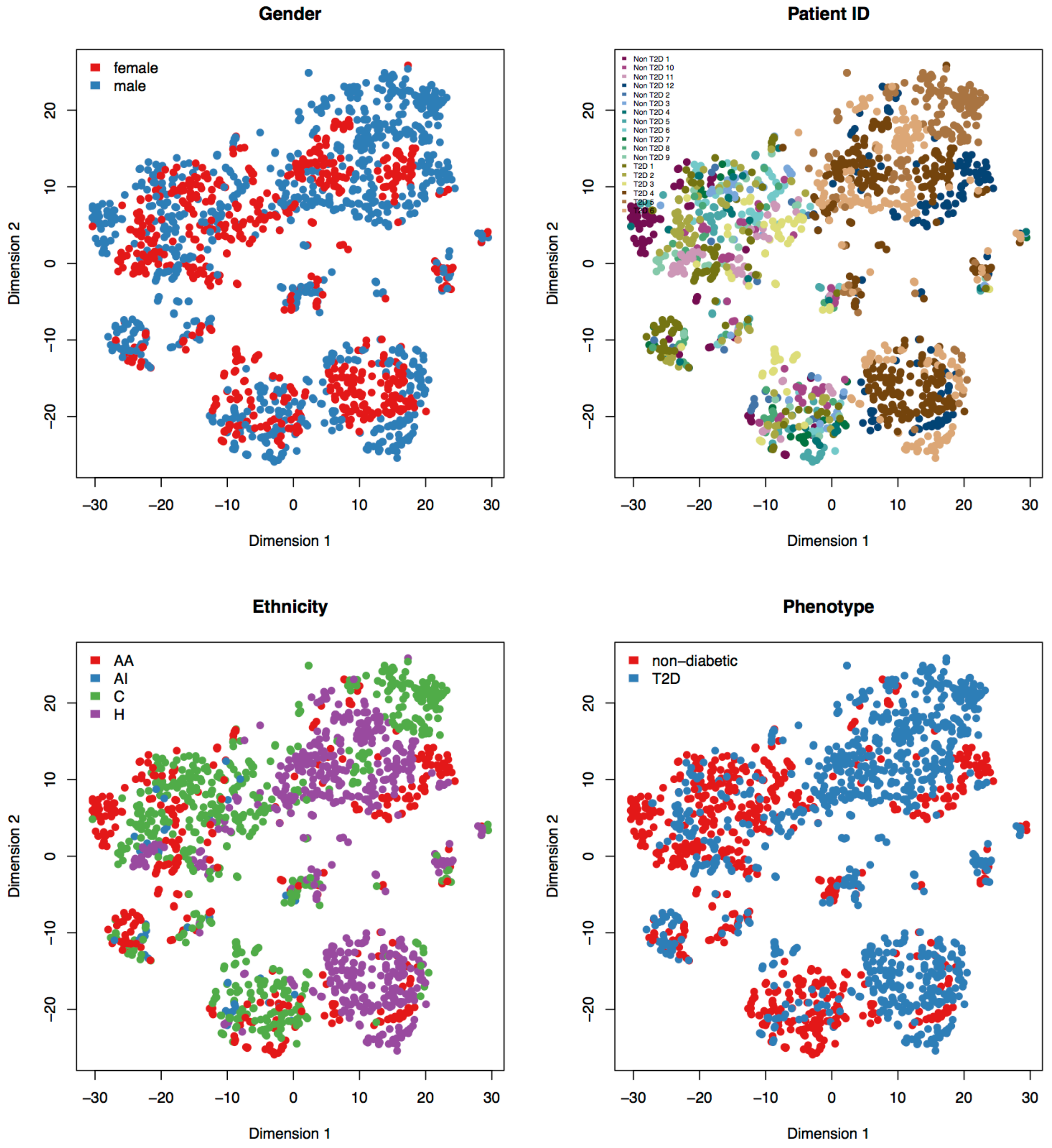

Figure S6. **Known variables explain single cell clustering and may confound with the heterogeneity stemming from different cell types.** tSNE plots generated using entire set of expressed genes are color-coded using known variables: sex, patient ID, ethnicity, and phenotype. Among these, patient ID and ethnicity drive the clustering of cells and can lead to misinterpretations of cell types.

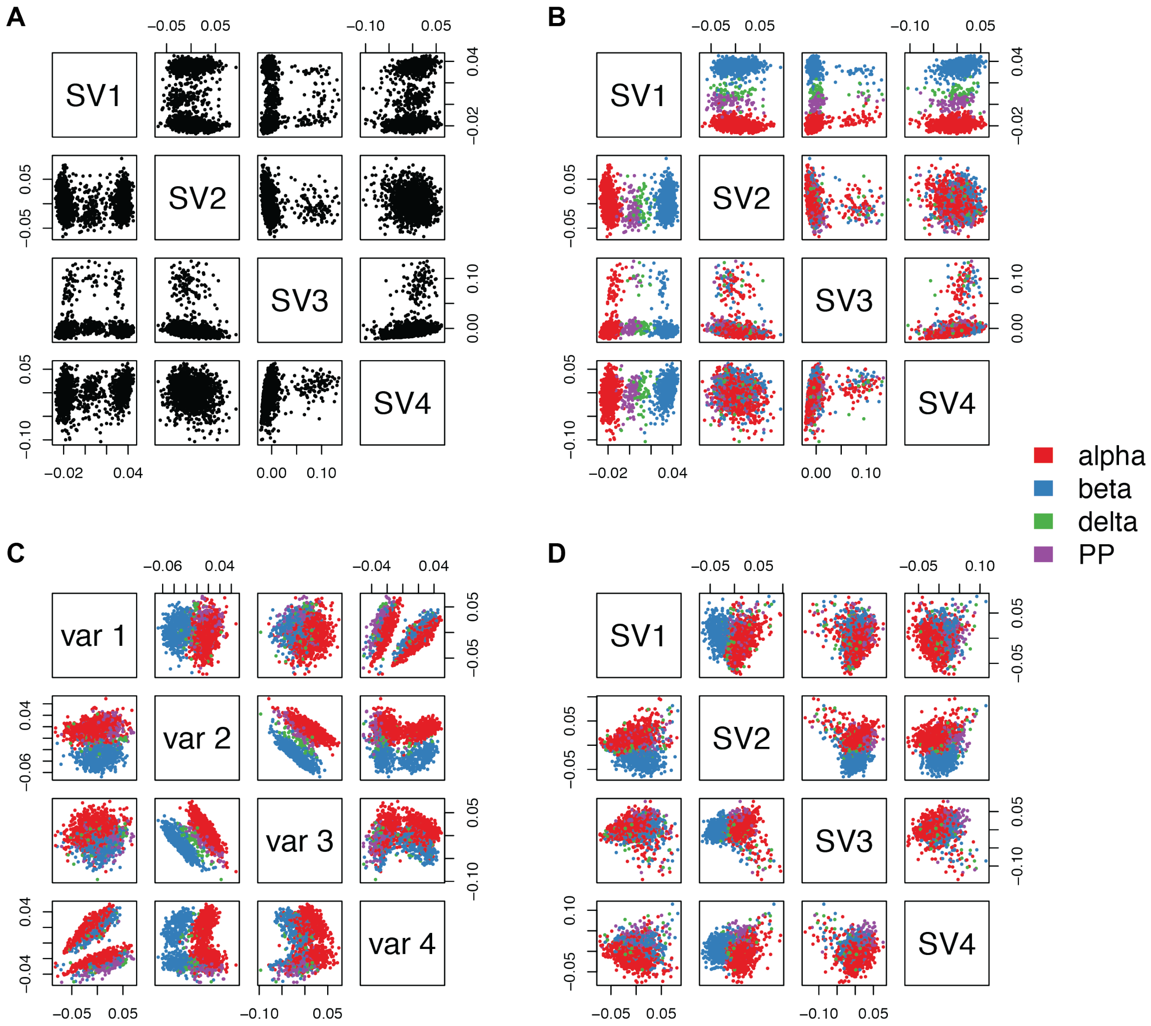

Figure S7. **IA-SVA effectively dissects the hidden variation in a second human islet scRNA-Seq data with strong confounders.** **(A)** Pairwise scatter plot of top four significant IA-SVA surrogate variables (SV). **(B)** Same as panel (**A**) where cell are color-coded with respect to original cell assignments. SV1 separate cells into disjoint clusters that matches to respective cell types as determined in the original study (see GO enrichment and pathway analyses results of 57 SV1 genes in Supplementary Table S5). SV3 captures technical heterogeneity stemming from stacked doublet cells (GO enrichment and pathway analyses results of 54 SV3 genes (FDR < 0.05 and $R^2 > 0.3$) are illustrated in Supplementary Table S7), which was observed in **Figure 2** and **Figure S3**. **(C)** Pairwise scatter plot of top four PCs from PCA on the same data. **(D)** Pairwise scatter plot of top four significant SVs obtained from USVA adjusted for all known factors that are also considered in the IA-SVA analysis (i.e., patient ID and geometric library size). IA-SVA outperforms alternatives in capturing hidden factors associated with cell types.

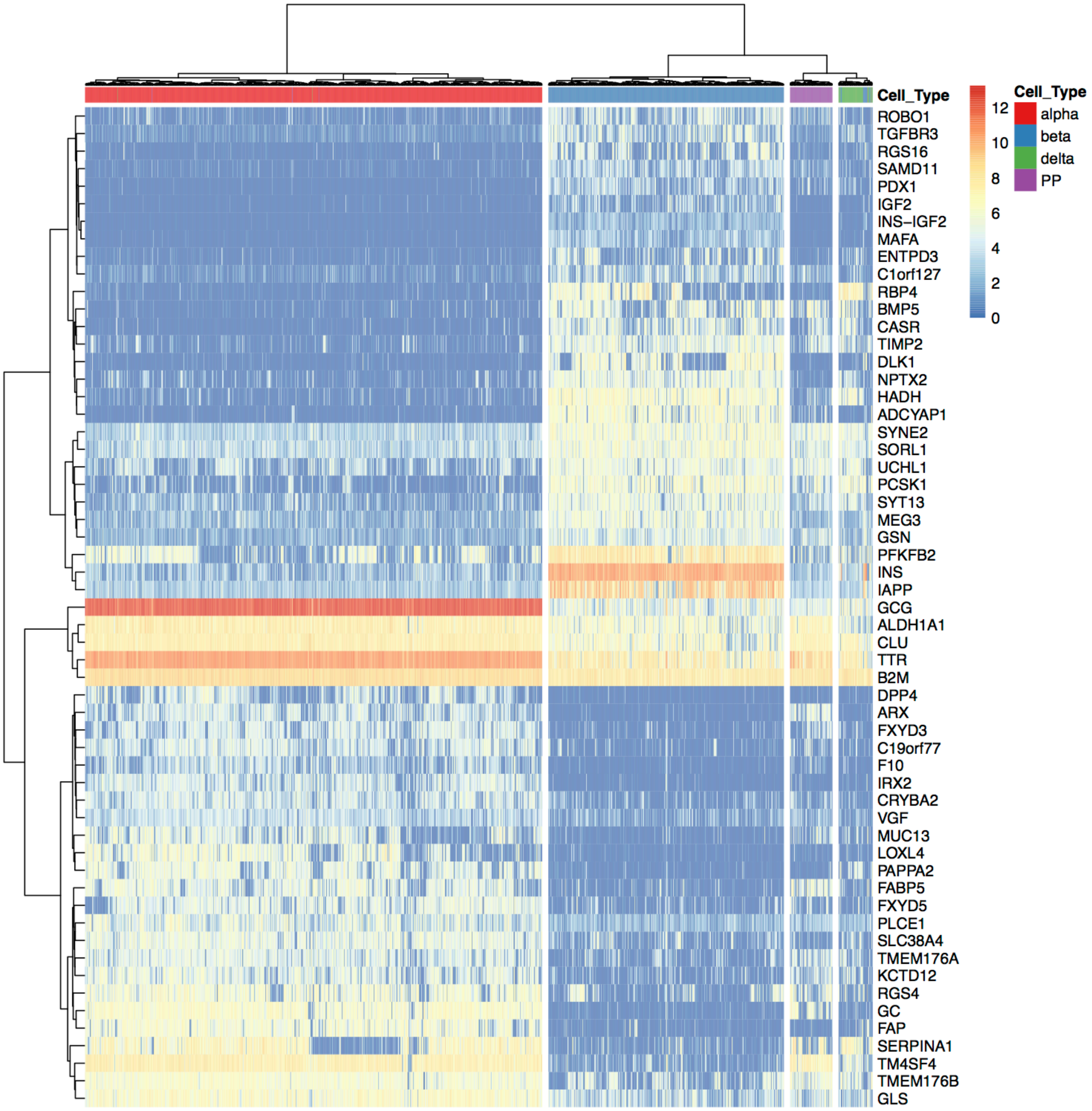

Figure S8. IA-SVA detects marker genes associated with different cell types among islet cells. Hierarchical clustering of islet cells using 57 marker genes detected by IA-SVA (ward.D2 and cutree_cols = 4). These genes are significantly associated (FDR < 0.05 and $R^2 > 0.5$) with IA-SVA's SV1. Note that cells are clustered together based on their cell types. Color-coding is based on the original study's assignments. The values in the heatmap and the color bar are log-transformed (base e) normalized counts (normalized by dividing each cell by its total counts then multiplying median of library size).

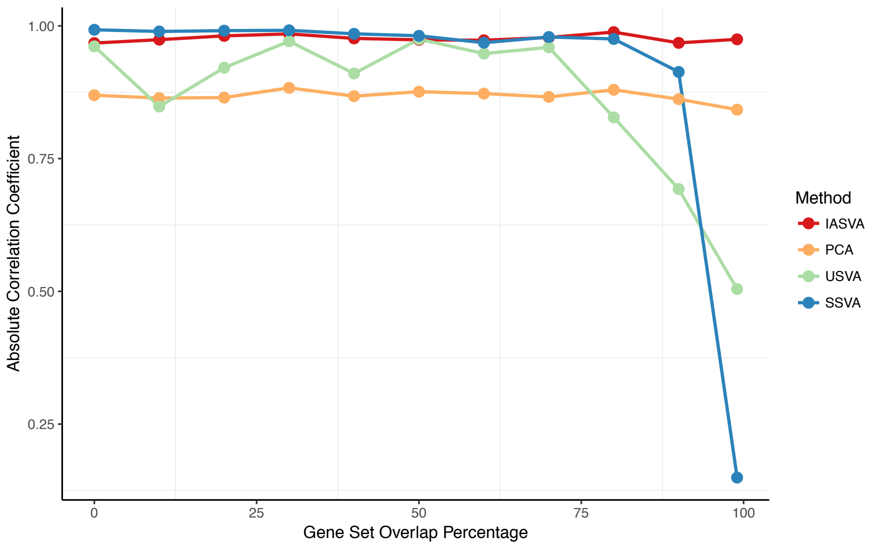

Figure S9. Accuracy of SVs detected when hidden and known factors affect overlapping sets of genes. We studied the performance of alternative methods in correctly inferring the stimulated SV when $k\%$, $k \in \{99, 90, 80, 70, 60, 50, 40, 30, 20, 10, 0\}$, of genes affected by the known factor are also affected by the simulated hidden factor (x axis). Note that, IA-SVA can still capture the hidden factor accurately even when there is 99% overlap between the gene sets. This performance is due to the fact that effect sizes for hidden factor can be larger for some of the overlapping genes in comparison to effect sizes for the known factor, which enables IA-SVA to identify these genes as marker genes of the hidden factor and infer the hidden factor effectively from these sets of genes.

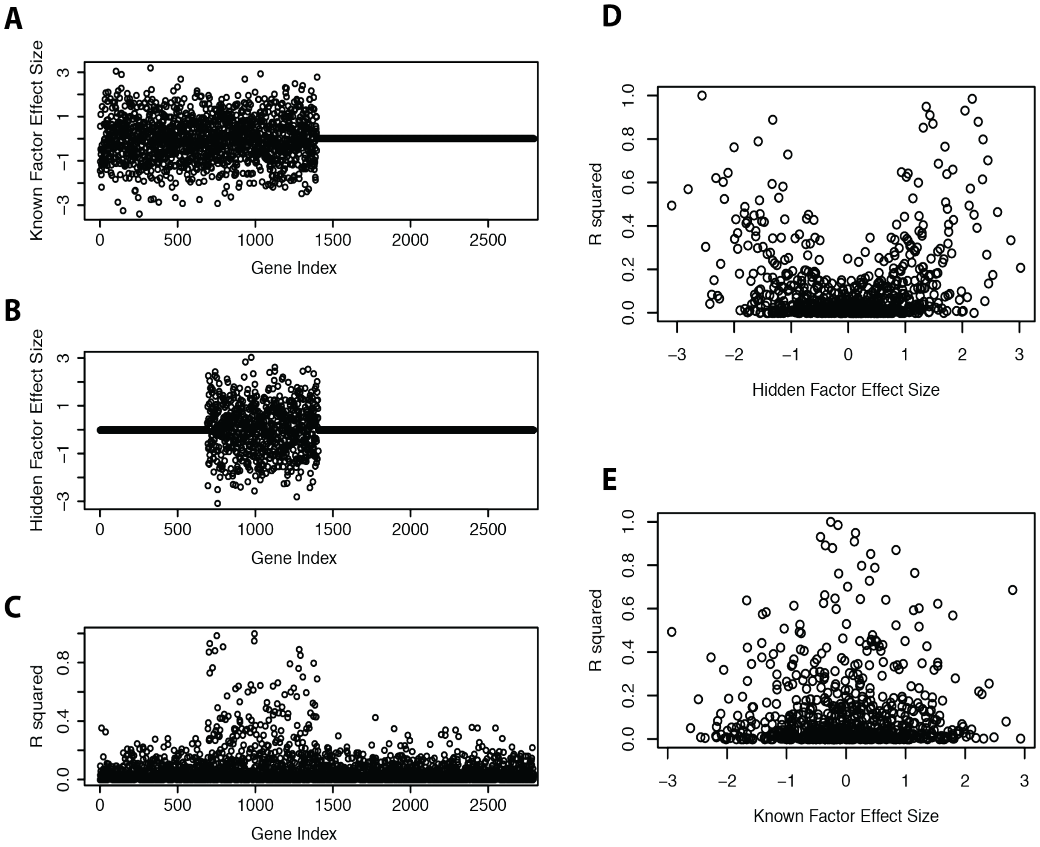

Figure S10. IA-SVA can still infer a set of genes significantly affected by the hidden factor even when the majority (99%) of the genes affected by the hidden factor are also affected by the known factor. We further studied simulation data generated under the extreme scenario (*k* = 99%, percent of genes affected by the hidden factor that are also affected by the known factor). (**A**) Effect sizes of the known factor on genes. (**B**) Effect sizes of the hidden factor on genes. (**C**) R^2 (gene weights) computed by IA-SVA per gene. Note that, IA-SVA can still capture genes strongly affected by the hidden factor under the extreme scenario (*k* = 99%). (**D**) R^2 of the genes affected by both factors as a function of the hidden factor effect size. Note that genes that have a high effect size also have higher R^2 values. (**E**) R^2 of the same gene set as a function of the known factor effect size. Among the genes affected by both factors, genes strongly affected by the hidden factor (genes with higher magnitude of effect size for the hidden factor) and weakly affected by the known factor (i.e., with lower magnitude of the effect size for the known factor) tend to have higher R^2 value (**D** and **E**). IA-SVA identifies these genes as the hidden factor marker genes and infers the hidden factor effectively from these genes.

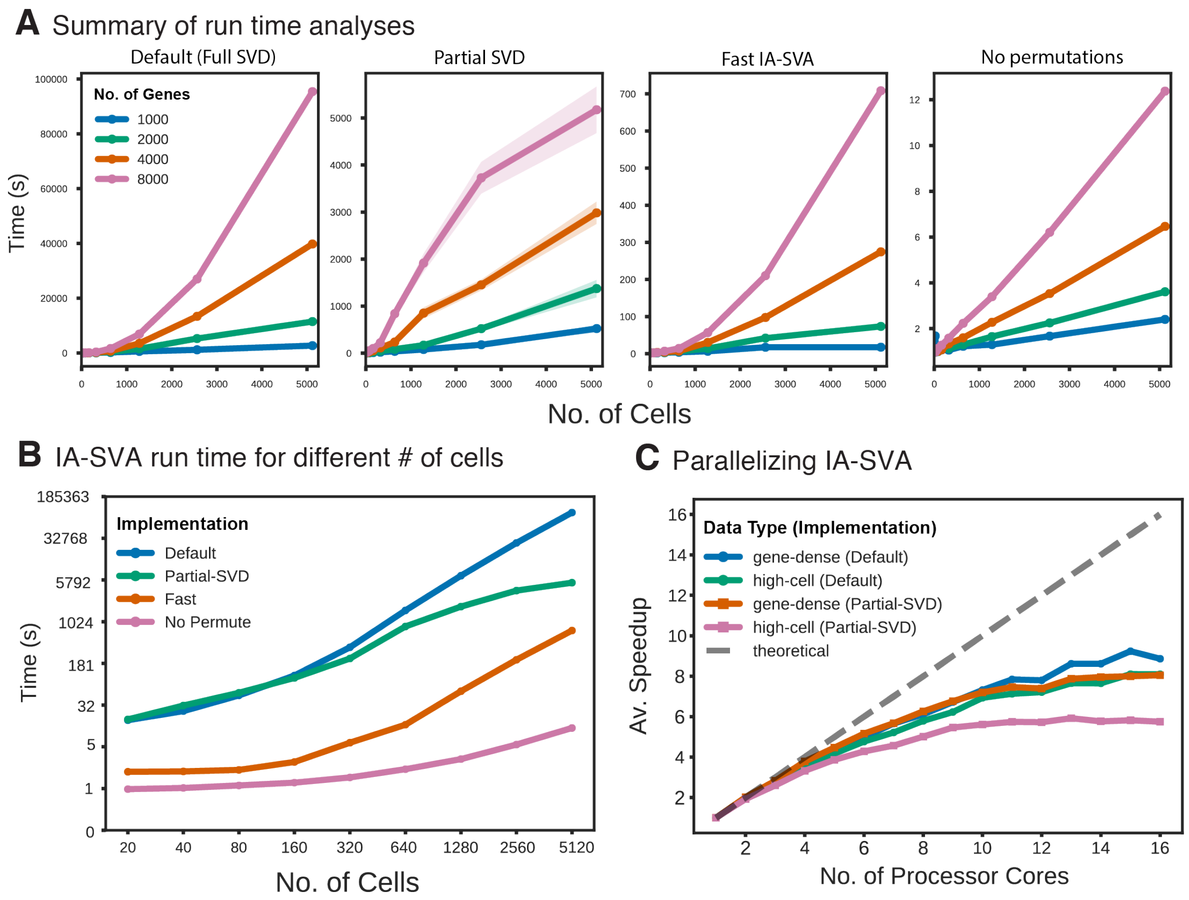

Figure S11. Empirical time complexity analysis for IA-SVA. (**A**) Run times for 4 different settings of IA-SVA for different number of cells and genes. Different colors represent different number of genes. Shaded regions depict standard error over 10 repeated runs. "Fast" and "No Permute" modes run much faster than modes that include permutations for significance testing. (**B**) Log-log plot comparing run time of alternative implementations of IA-SVA as a function of number of cells (the number of genes is fixed for 8000). For datasets with many cells, Partial-SVD mode substantially decreases the amount of time used compared to the Default (full SVD) mode. Without the significance testing of surrogate variables, Fast mode runs in a few minutes even for large datasets. IA-SVA's run time is approximately quadratic in time for Default and Fast modes, while being linear for No Permute and Partial-SVD modes when increasing the number of cells. It is approximately linear in time for all modes when increasing the number of genes (plot for number of genes not shown). (**C**) Average speedup of IA-SVA's run time vs. number of processors used. IA-SVA analyses were performed for 2 data set sizes: a gene-dense dataset (16000 genes × 640 cells, e.g., Fluidigm C1) or a cell-dense dataset (1000 genes × 5120 cells, e.g., 10X Genomics). From these plots, Amdahl's law suggests that approximately 90-95% of runtime is parallelizable. All time complexity analyses were conducted using HP Proliant SL/XL Series servers with 2.5-2.6GHz processor clock speed and 256 GM RAM.

Text S1. Rationale behind IA-SVA's weighting schema

Here, we further describe in more details how IA-SVA in particular the R^2 weighting strategy works. For this, let's assume a simple gene expression model for gene i affected by two factors—one known and one hidden:

$$y_i = \beta_i x_i + \delta_i z_i + \varepsilon_i$$

, where y_i is the gene expression vector of gene i, x_i and z_i are the known and hidden factors, respectively, β_i and δ_i are effect sizes of the corresponding factors and ε_i is the error term vector. Let's assume that the known and hidden factors are strongly correlated and their effect sizes (β_i and δ_i) are randomly distributed across affected genes. Under this model assumption, we can classify genes into four different groups based on the effect of the two factors on expression levels:

Group 1: Genes strongly affected by the known factor and weakly or not affected by the hidden factor (i.e., $|\beta| \gg |\delta|$)

Group 2: Genes weakly affected (or not affected) by the known factor but strongly affected by the hidden factor (i.e., $|\beta| \ll |\delta|$)

Group 3: Genes affected by both known and hidden factors with similar effect sizes (i.e., $|\beta| \approx |\delta| > 0$)

Group 4: Genes affected by neither the known factor nor the hidden factor (i.e., $|\beta| = |\delta| = 0$)

Using the R^2-based weighting strategy, we aim to identify Group 2 genes, which are strongly affected by the hidden factor but weakly affected or not affected by the known factor. In Step 2 of IA-SVA, the PC1 is obtained from the residual matrix $Y'_{m\times n}$, hence the known factor is stripped out. Therefore, PC1 is implicitly orthogonal to the known factor, hence would provide a biased estimate of the hidden factor when the known and hidden factors are correlated, therefore PC1 should not be directly used as an estimate for the

hidden factor if the goal is to accurately estimate the hidden factor. However, our algorithm still (similar to SVA) assumes that the hidden factor contributes significantly to the variation in PC1 (the first principal component explaining the largest proportion of the variation in the residual matrix) and thus the hidden factor strongly correlates with the PC1. Therefore, we hypothesize that PC1 can still be useful to sort genes based on their strength of association (i.e., R^2) with the hidden factor. Intuitively, genes that belong to Group 1 and Group 3 will have low R^2 because the effects of the hidden factor on these genes will be removed when the known factor is regressed out. Group 4 genes will also have low R^2 because these genes were not affected by both factors to start with. However, Group 2 genes, since they are strongly affected by the hidden factor, will have high R^2 even though the effects of the hidden factor on the genes will be diminished when we regress out the known factor. As shown in Figure S10, genes strongly affected by the hidden factor and weakly affected by the known factor tend to have higher R^2 values (Figures S10D and E). Using this property, IA-SVA focuses on identifying Group 2 genes based on their high R^2 values. These genes behave similar to the 'negative control genes' (genes known a priori not to be affected by the known factor, i.e., these genes include Group 2 and Group 4 genes) used in the RUV-2 method[1]—when negative control genes are known a priori, one can estimate the hidden factor very accurately even if the known and hidden factors are highly correlated. Our weighting schema highlights Group 2 genes (Step 5), hence the hidden factor explains the largest proportion of variation in read counts matrix of Group 2 genes (the weighted log-count matrix $Y''_{m\times n} = Y_{m\times n}W_{n\times n}$). Therefore, at this step (Step 5) regressing out the known factor again is not required—we simply conduct SVD on $Y''_{m\times n}$ to extract only PC1 and use it as the hidden factor estimate (Step 6). We repeat this procedure to detect further unknown factors, this ensures that every round we can detect a new set of Group 2 genes, which could be representing different sources (e.g., cell cycle vs. cell state). These are the major differences of IA-SVA methodology. Identifying all Group 2 genes will be difficult (Figures S10B and C). However, we observed that IA-SVA can infer the hidden factor accurately using a subset of Group 2 genes with high R^2 (e.g., > 0.3), which is sufficient enough to estimate the hidden factor.
